# Unraveling the Impact of Gut and Oral Microbiome on Gut Health in Inflammatory Bowel Diseases

**DOI:** 10.3390/nu15153377

**Published:** 2023-07-29

**Authors:** Hala Elzayat, Ghaidaa Mesto, Farah Al-Marzooq

**Affiliations:** 1 Department of Medical Microbiology and Immunology, College of Medicine and Health Sciences, United Arab Emirates University, Al Ain 15551, United Arab Emirates; 2 Zayed Center for Health Sciences, United Arab Emirates University, Al Ain 15551, United Arab Emirates

**Keywords:** inflammatory bowel disease, Crohn’s disease, ulcerative colitis, gut microbiome, oral microbiome

## Abstract

Inflammatory bowel disease (IBD) is a complex disorder characterized by chronic inflammation of the gastrointestinal tract (GIT). IBD mainly includes two distinct diseases, namely Crohn’s disease and ulcerative colitis. To date, the precise etiology of these conditions is not fully elucidated. Recent research has shed light on the significant role of the oral and gut microbiome in the development and progression of IBD and its collective influence on gut health. This review aims to investigate the connection between the oral and gut microbiome in the context of IBD, exploring the intricate interplay between these microbial communities and their impact on overall gut health. Recent advances in microbiome research have revealed a compelling link between the oral and gut microbiome, highlighting their pivotal role in maintaining overall health. The oral cavity and GIT are two interconnected ecosystems that harbor complex microbial communities implicated in IBD pathogenesis in several ways. Reduction in diversity and abundance of beneficial bacterial species with the colonization of opportunistic pathogens can induce gut inflammation. Some of these pathogens can arise from oral origin, especially in patients with oral diseases such as periodontitis. It is essential to discern the mechanisms of microbial transmission, the impact of oral health on the gut microbiome, and the potential role of dysbiosis in disease development. By elucidating this relationship, we can enhance our understanding of IBD pathogenesis and identify potential therapeutic avenues for managing the disease. Furthermore, innovative strategies for modulating the oral and gut microbiome can promote health and prevent disease occurrence and progression.

## 1. Introduction

Inflammatory bowel diseases (IBDs) are chronic idiopathic disorders with relapsing inflammation of the gastrointestinal tract (GIT). More than six million patients are suffering from IBD, and more cases are diagnosed worldwide [1]. In the 20th century, IBD was not prevalent in Asia, Africa, Eastern Europe, or South America but was more common in North America and Europe. Therefore, IBD has been recognized as a Western disease in the past [2]. Nowadays, IBD incidence and prevalence have significantly grown all over the world, spreading to all continents.

IBD includes two main types, which are Crohn’s disease (CD) and ulcerative colitis (UC). There are several distinctions between CD and UC despite the similarities between the symptoms of these two diseases [3]. While UC predominantly affects the colon and rectum, CD affects mostly the terminal ileum and the large intestine, with transmural inflammation involving any part of the GIT from the oral cavity to the anal tract [1]. The symptoms of IBD may be mild to severe, intermittent with a flare-up period [4]. Although recurrent diarrhea, abdominal discomfort, gastrointestinal bleeding, and weight loss are the predominant symptoms of IBD, many patients also experience extraintestinal problems, often in the form of oral, skin, joint, eye, and bone lesions. It is interesting to note that oral lesions can occasionally occur months or years before intestinal signs [5]. In children, the disease is more severe and characterized by rapid progression, more extraintestinal involvement, and the need for surgery in addition to the obvious retardation of growth and puberty [6]. Children with IBD may respond to therapy more favorably than adults. Inflammation should be treated early on in the course of the illness in order to avoid long-term complications including strictures, blockage, the need for surgery, and hospitalization [7].

In recent years, the captivating topic of the microbiome and its profound significance for human health has gained much attention. Research on the human microbiome has expanded rapidly, aided by advancements in culture-independent technologies such as metagenomics, transcriptomics, metabolomics, and proteomics. Thus, microbiota functional dynamics, interaction with the host, and contribution to the pathophysiology of diseases have been explored comprehensively using these multi-omics techniques [8]. Imbalance or disruption of the microbiome has been related to various health conditions, including obesity, diabetes, mental disorders, and IBD, among others [9]. The precise etiology of IBD is not fully understood; however, emerging evidence suggests that alterations of GIT microbiota play a significant role in the development and progression of IBD [10]. Recently, the oral microbiome has gained more attention in relation to many diseases [11], including IBD [12]. It is still debatable whether the alterations of microbiota represent the cause of IBD or if they happen as a consequence of the disease’s pathological changes [13].

In this review, we delve into the gut and oral microbiome, their connections, and the detrimental effects of their dysbiosis on IBD pathogenesis and progression. Furthermore, we shed light into the potential avenues for restoring the microbiome to treat IBD.

## 2. Overview of the Significance of Normal Microbiome

The microbiome refers to the set of microorganisms residing at a particular niche and working together in a mutualistic relationship with the human host [14]. The human microbiome consists of trillions of microbial cells inhabiting various parts of the body. Most of the microbiota present in the human body are concentrated in the GIT. The human digestive system microbiome is composed of hundreds of bacterial and fungal species, and these microorganisms have 150 times more genes than the human genome [15]. The gut microbiota, the body’s richest reservoir of bacteria, coexists with its host in varying densities throughout the GIT, peaking in the colon with up to 10^12^ bacteria/g of gut luminal contents [16]. The four major bacterial phyla that are most frequently seen (90%) are *Firmicutes*, *Bacteroidetes*, *Proteobacteria*, and *Actinobacteria*, with 500–1000 different species residing in the gut. Intestinal microbes can be classified into aerobic, anaerobic, and facultative anaerobic bacteria according to aerobiosis or anaerobiosis. Anaerobic bacteria are the most prevalent intestinal bacteria. The distal parts of the small intestine and colon are where these microbes are most found. The majority of these bacteria are attached to the intestinal mucosa, adherent to the epithelial cells to create a bacterial layer, which eventually has an impact on how well the intestinal system works [17].

The gut microbiome has a pivotal role in maintaining overall health and well-being. This ecosystem influences various physiological processes, including digestion, metabolism, absorption of nutrients, and production of vitamins. It is also involved in immune system development and regulation, ensuring its effective response to pathogens while preventing excessive inflammation [18]. Furthermore, the gut microbiome acts as a protective shield, preventing harmful microorganisms from colonizing and causing infections. It protects the host against bacterial infections by competing with pathogens for space and nutrition and by creating antimicrobial peptides, such as bacteriocin, as well as hydrogen peroxide [8]. Furthermore, microbiota generate a variety of metabolites, such as short-chain fatty acids (SCFAs), specifically butyrate, acetate, and propionate, from dietary products. These metabolites influence host health and physiological functions and have a significant effect on gut barrier function and immune responses [19].

Overall, host physiology is impacted significantly by the composition, function, and metabolites of gut microbiota.

## 3. Dysbiosis and Relation to Diseases

**Figure 1:**
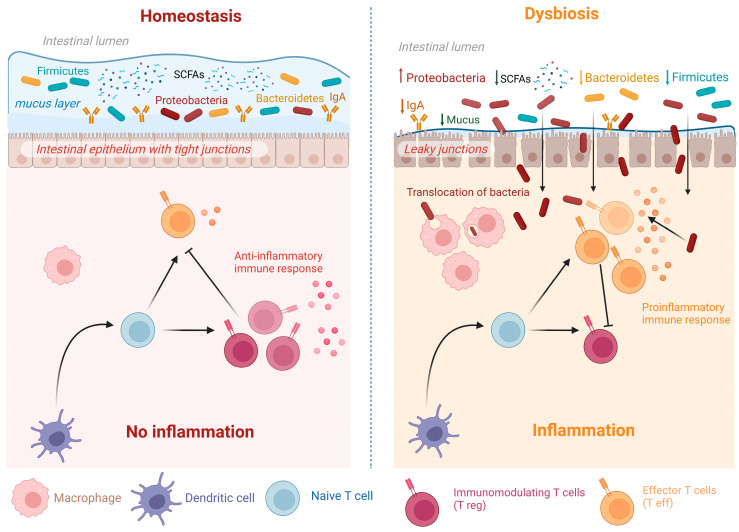
Dysbiosis versus homeostasis. GIT homeostasis is the balance between the functions of the cells in the epithelial lining of the gut and the immune responses to microbes whereby beneficial microbiota predominate, with tolerance of commensal bacteria residing on a healthy epithelium and intact tight junctions. In this case, inflammation is suppressed by the action of immunomodulatory T cells (regulatory T cells; Treg), which counteract the action of proinflammatory T cells (effector T cells). The reverse is true in the case of dysbiosis. Mucus layer is lost, and epithelial cells’ tight junctions are damaged and become leaky to harmful microbiota, which interact with immune cells to induce inflammation.

If the normal microbiome is essential for maintaining suitable health, indeed, its disturbance is harmful [20]. Dysbiosis refers to an imbalance or disruption in the microbial abundance and/or function caused by alterations in microbiota composition and diversity in a particular ecosystem, such as the GIT [21]. Noteworthy, dysbiosis is a complex and multifactorial phenomenon, and its contribution to various diseases is still being actively researched. Research has shown that dysbiosis has significant implications for health. The disrupted microbiota may lead to a loss of beneficial functions performed by the microbes, impairing the metabolism of nutrients, affecting the intestinal barrier integrity, and altering the functions of innate and adaptive intestinal immunity compromising immune system regulation [18]. This can result in a broad array of diseases, including but not limited to metabolic disorders and gastrointestinal and inflammatory conditions [8,20]. Figure 1 summarizes the changes that occur in dysbiosis compared to normal homeostasis and the contribution of microbiota to both processes.

As shown in Figure 1, dysbiosis can cause disturbance of intestinal mucosal homeostasis. These changes are driven by several intrinsic and extrinsic factors, collectively and collaboratively leading to dysbiosis. Among these factors are genetic predisposition, diet, lifestyle, ecological factors, and therapeutic interventions such as antibiotics, lipid-lowering drugs, laxatives, proton pump inhibitors, ACE inhibitors, beta blockers, antidiabetic agents such as metformin, and others [22]. Furthermore, several studies reported that dysbiotic microbiota can affect human response to therapeutic intervention. The best example is microbiota influence on cancer immunotherapy, as the gut microbiome was shown to have the potential to modulate the efficacy and toxicity of several antitumor drugs against different types of cancer, thus impacting the outcome of such therapies [23]. In the coronavirus disease 2019 (COVID-19) era, some studies reported that gut microbes influence the immune response to severe acute respiratory syndrome coronavirus 2 (SARS-CoV-2) infection. Increased susceptibility to COVID-19 with more severe symptoms was noted among patients with dysbiotic gut microbiota [24,25]. This was explained by the ability of SARS-CoV-2 to replicate in the enterocytes, interacting with the gut microbiota, which in turn affected host immune responses. On the other hand, COVID-19 itself caused dysbiosis in infected patients [26], with persistent microbiota perturbations even after the clearance of infection [25].

Taken together, dysbiosis not only predisposes to diseases, but also impacts host’s response to infections, and medications. Thus, microbiome homeostasis is essential for suitable health while dysbiosis has a detrimental effect on health.

## 4. Gut Microbiota Alterations in IBD and Relation to IBD Pathogenesis

Inflammatory bowel illness is frequently a polygenic condition involving barrier dysfunction, gut microbiota, and dysregulated host responses to microbial stimulation. The precise etiology and pathogenesis of IBD remain unclear. The most accepted hypothesis explaining IBD is that chronic intestinal inflammation coupled with aberrant immune responses are derived from complex interactions between genetics, environmental factors, and the host immune system [27]. IBD is genetically connected to host pathways that suggest an underlying role for abnormal immune responses to gut microbiota [28]. The initiating stimuli for immune dysregulation are not fully explained by genetic predisposition. Genetic loci associated with the development of IBD can alter the delicate relationship between the microbiota in the GIT and the host, leading to a dysregulated immune response and intestinal inflammation [29]. In a meta-analysis of IBD genome-wide association in more than 75,000 cases and controls, many genes associated with IBD were found responsible for immune system recognition of microbes, suggesting that these patients are unable to either nurture beneficial bacteria or destroy the harmful ones [30].

Recently, deep-sequencing analyses of the microbiota in IBD patients have been instrumental in proving the strong association between dysbiosis and IBD development [20], but more research is indeed needed to identify the key species leading to IBD or contributing to the activity of the disease. In healthy people, the gut microbiome is highly individualized in both its condition and its dynamics. Nevertheless, cross-sectional research has shown that IBD is associated with gut microbiota dysbiosis [31]. In a previous study, Illumina HiSeq 2000 platform was used for the sequencing of the V4 amplicons [32]. By sequencing 248 million 16S rRNA gene amplicons, the microbiome composition in each sample was identified. Research has shown that individuals with IBD have distinct microbial profiles and has shown that IBD patients’ gut microbiota considerably varied from microbiota inhabiting the gut of healthy individuals.

Additionally, many identified microbial metabolites are reduced in people with IBD compared to healthy people, according to metabolomic studies. Research has speculated that IBD can be caused by the increase in certain proinflammatory bacterial metabolites and their producing species in IBD patients with the depletion of other anti-inflammatory metabolites and associated species that might have a protective role [33]. SCFAs are produced by *Firmicutes* and *Bacteroidetes* from carbohydrates, which triggers the production of associated proteins necessary for the development of tight junctions and supports intestinal mechanical barriers. Moreover, polysaccharide A generated by *Bacteroidetes* has anti-inflammatory effects by induction of Treg cells to release interleukin-10 (IL-10) which has a protective role on the intestinal barrier. However, the intestinal barrier function is destroyed and IBD vulnerability is increased because these microbiota are depleted in the colon of IBD patients [34]. Furthermore, research in germ-free mice indicates that gut bacteria impact body fat accumulation, metabolism, and immunological function [16]. The function of the microbiome in the pathogenesis of IBD is still being contested; however, the illness involves a significant inflammatory response that can be induced by acquired infection or alterations in the host’s own microbiome [35].

The mucosal layer in the gut is composed of a mucin complex that is rich in O-glycosylation. It has an imperative role in controlling germs growth, in addition to provision of food and space for intestinal microbiota to live. The stratification of the mucus layers and mucin synthesis are both influenced by gut bacteria (Figure 1). Any anomalies could lead to a malfunction in the gut population, which would lead to inflammation-related harm [36].

Bacterial 16S rRNA gene sequencing has improved our understanding of the microbiota makeup in various regions of the human body as well as the complex bacterial communities in IBD. Next-generation sequencing technologies helped in the precise identification of gut microbiota alterations in CD patients. An overabundance of bacteria from *Enterobacteriaceae* has been observed in individuals with IBD. On the other hand, beneficial bacteria like *Faecalibacterium prausnitzii*, which produces anti-inflammatory substances [37], are often depleted in individuals with CD. Gut dysbiosis, which includes enrichment of *Proteobacteria*, depletion of *Firmicutes* and *Bacteroidetes*, and a reduction in gut biodiversity, is definitely associated with CD [5]. Studies have pointed to the possibility of having certain infections implicated in CD pathogenesis, such as infections caused by *Mycobacterium avium* (subspecies paratuberculosis) and infections with adhesive-invasive strains of *E. coli*. These infections are most likely causing an imbalance in the total microbiota community, which is more significant for CD development [16]. Another intriguing finding is that the gut microbiota in CD patients is more unstable than in healthy persons. However, it is unclear whether the observed dysbiosis is the cause or the result of intestinal inflammation in CD, with growing evidence supporting the hypothesis that both hereditary and environmental variables are directly connected to dysbiosis [38]. In a recent Chinese cohort, patients with a recent diagnosis of CD possessed gut microbiota deficient in SCFAs-producing bacteria, such as *Clostridium IV*, *Fusicatenibacter*, *Coprococcus*, *Blautia*, and *Dorea*, with enrichment of Gram-negative pathogens such as *Proteus*, *Escherichia*, and *Shigella* when compared to healthy controls [39]. There was a significant correlation between the levels of some of these genera and various clinical parameters, inflammatory indicators such as fecal calprotectin, C-reactive protein, erythrocyte sedimentation rate, and counts of gland and goblet cells. These findings are important as they reflect microbiota perturbation early during the course of the disease with a progressive increase in the abundance of opportunistic pathogens. The latter can exacerbate microbiome dysbiosis in CD patients, hence, impacting the disease progression and outcome.

As for UC, alterations in gut commensals involved in microbiota-mediated defenses have been reported. When 16S rRNA microbial profiling was used for the characterization of the microbiota in UC patients, a significant decrease in *Bifidobacteria* was noted, especially the species *Bifidobacterium bifidum*, suggesting that this taxon may be biologically involved in UC pathogenies [40]. As for CD, the complex pathogenesis of UC is thought to be influenced by the interactive effect of environmental, genetic, and microbial variables. Mucosal permeability rises in UC due to an excessive inflammatory response within the lymphoid tissues. There is still much to learn about gut microbiota and its composition in UC, including various phyla such as *Bacteroidetes*, *Firmicutes*, *Actinomycetes*, and many others. UC status had been linked to microbial dysbiosis with observed temporal variations in microbiota composition as reported by multiple genomic-based microbiome studies. Although new initiatives went beyond genomics to profile the microbiota in UC, it is still vague whether these changes are causal or associative in nature [41]. To fill this knowledge gap, the host–microbiota interactions were examined in a big cohort of IBD patients to compare the metagenomic-metaproteomic strategy to more traditional 16S rRNA gene amplicon sequencing, fecal metabolomics, and serum proteomics techniques. Multiple bioinformatic tools as QIIME, DIAMOND, and STAMP were used to conduct a thorough, unbiased meta-analysis of the gut microbiome data from five distinct cohorts of IBD patients who originated from five different countries. Similar microbial species depletion in CD patients was verified by the analysis. It did, however, show a divergent enrichment in the UC cases. Following a thorough analysis, multi-omics data revealed that some proteases from the genus *Bacteroides* may play a role in UC pathogenesis. Meta-omics data specifically identified *Bacteroides vulgatus* proteases as possible treatment targets for UC [41]. Notably, butyrate, an essential SCFA made by some species, such as *F. prausnitzii*, was found to be increased in UC patients. This increase may signify an adaptive enrichment due to the vital role of butyrate in maintaining the epithelial cells in the gut [42]. In the induced UC model, intestinal microecology is significant, and the composition of the gut microbiome varies rapidly during the onset and progression of the disease. Researchers have demonstrated that *Lactobacillus* abundance decreased in UC. Anaerobic probiotic *Lactobacillus* produces vitamins and amino acids in the intestinal system. It may induce regulatory T cells (Treg) differentiation, reduce proinflammatory cytokines secretion, stop the growth of pathogenic organisms, and prevent the release of endotoxins, in order to balance the intestinal immune system and stop the progression of UC [43]. *Saccharomycetales* enrichment with microbiome diversity depletion was demonstrated as typical of UC gut dysbiosis using both fungal internal transcribed spacer 2 sequencing and bacterial 16S rRNA sequencing of fecal samples from 30 UC compared to 13 healthy controls [44]. Aside from that, it was discovered that four unique microbial community states (MCSs) verified their presence in a separate UC cohort and showed how they were related to the ethnicity of the patient as well as the severity of the illness [44]. Clinical, endoscopic, radiological, and histological results were used to make the diagnosis of UC. Through the sequencing of the V3-V4 region of the 16S rRNA gene and with the use of bioinformatic techniques, the makeup of the gut microbiota was identified. The functional makeup of the gut microbiota was anticipated by the Phylogenetic Investigation of Communities by Reconstruction of Unobserved States (PICRUSt) program. Intestinal flora in UC patients was considerably less diverse and rich than in healthy control subjects. When UC patients with active disease were compared to patients in remission status, alpha diversity did not show statistically significant variations, although intestinal flora compositions varied noticeably based on beta diversity analysis [45].

Comparatively to UC, the genetic effects of CD on the microbiota have received more attention, particularly those involving the NOD2 gene [15]. However, investigations involving mice and humans have also been conducted to shed light on UC variations connected to the microbiota. MHCII, HLA, ATG16L1, LRRK2, CARD9, CLEC7A, MUC5AC, and cytokine-related pathways (IL-17, IL-22) are specifically implicated in UC. Since this may be the only component not shared with CD, changes in the expression of mucus-related variations, including MUC5AC, seem relevant to determining peculiarities of UC development. As a result, this characteristic draws attention to the potential connections between microbiota dysbiosis, mucus composition, epithelial barrier disruption, and the development of UC [46]. Indeed, more research is needed to elucidate these links in IBD patients.

## 5. Oral–Gut Axis and the Role of the Oral Microbiome in IBD Pathogenesis

Although the mechanisms of the gut microbiota interaction with the immune system have received considerable attention [47], little is known about the connection between the oral microbiota and host immunity in IBD, and much less is known about IBD-related oral microbiota dysbiosis [47]. It is important to consider that the oral microbiome ranked as second after the gut microbiome in terms of having huge number of species, with high diversity and complexity of the microbial community [48]. The complex microbiota that inhabits the mouth includes more than 700 prominent species and contributes greatly to the host’s oral and extra-oral health. The oral and gut microbiomes, contain most of the microbial biodiversity in the human microbiome. However, these two groups are significantly different in terms of makeup. The most dominant bacteria in the oral cavity are facultative, sugar fermenting organisms such as *Streptococcus* and *Actinomyces*, whereas the most dominant organisms in the gut are metabolically varied population of anaerobic bacteria such as *Clostridium* and *Bacteroides*. *Bacteriodetes*, *Firmicutes*, *Proteobacteria*, *Synergistetes*, *Fusobacteria*, *Spirochaetes*, *Actinobacteria*, *SR-1*, and *TM-7* are among the most prevalent bacterial phyla discovered in the oral cavity, using the human oral microbe identification microarray [47]. Surprisingly, the oral microbiome has less interindividual variance than the gut microbiome. The increased interindividual variance reported in gut microbiomes seems to be connected to the larger influence of variables such as nutrition and antibiotic use, whereas the oral microbiome appears to be more robust to similar challenges [47].

Since the GIT and the oral cavity are one continuous system, oral health may be directly related to gut health. Recent studies have shed light on the role of the oral microbiome in shaping the gut microbiome and subsequently impacting gut health. This is not surprising, as shown in the Human Microbiome Project, whereby oral and stool microbiota overlapped in approximately half of the subjects [49]. Some studies showed that the oral microbiota can be translocated from the mouth into the intestines causing microbial dysbiosis and inflammation but other studies on the other hand suggested that the oral microbiota dysbiosis will occur as a consequence of the gut inflammation so that it can be used as a biomarker of IBD [50]. Oral bacteria can enter the digestive system through swallowing, aspiration, or translocation, thus potentially influencing the microbial ecosystem in the gut [51]. Microorganisms may pass from the oral cavity to the gut while still being alive, despite the barrier of the gastric acidic pH, suggesting that oral–gut transit of microbes is likely common [50]. Pathological alterations of the oral microbiota, such as changes caused by periodontal disease, are associated with multiple inflammatory disorders, including IBD [52]. Inflammatory processes in the oral cavity can release inflammatory mediators that may affect the gut’s immune response and exacerbate intestinal inflammation. Furthermore, ulcerous periodontal pockets can serve as a portal of entry for subgingival microbiota with their components into circulation, leading to low-grade systemic inflammation, which can induce various systemic diseases [53].

**Figure 2:**
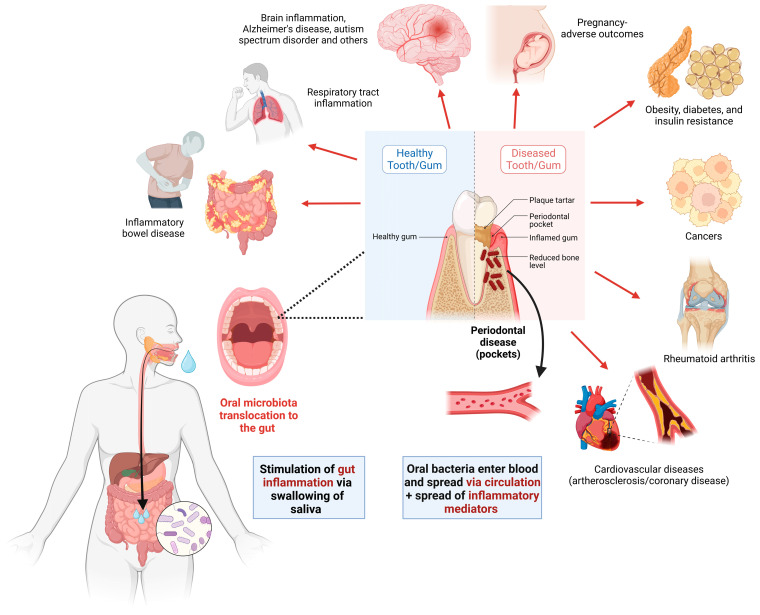
Oral microbiota translocation. Oral bacteria can reach the gut through swallowing of saliva loaded with the oral microbiota, which can induce gut inflammation. It can also enter the blood via ulcered gingival tissues if the person is having periodontal disease. Systemic spread of the oral microbiota and their interaction with the immune system can lead to inflammatory changes and contribute to the pathogenesis of many diseases (examples are shown in the figure).

Figure 2 shows oral microbiota translocation methods, impact on the systemic health, and relation to various diseases, including IBD.

Recent studies suggest crosstalk between gut and oral microbiomes, which may also contribute to IBD pathogenesis [52]. Metagenomic studies within the last 10 years have revealed comprehensive information on microbiome dysbiosis and surprisingly found a higher abundance of common oral taxa (such as *Veillonella*, *Haemophilus*, and *Eikenella)* in the gut of IBD patients, while many bacteria producing SCFAs were reduced. Microbiota translocation from the mouth to the gut may be a unique phenomenon leading to microbial dysbiosis, which is a special signature of IBD [50]. Pathobionts can move between the mouth and gut following a local inflammation starting in the oral cavity, then disseminating to the intestine [54]. A recent study in mice has shown that inflammation of the oral mucosa combined with oral pathobionts’ proliferation led to colitis via gut colonization and the induction and migration of bacteria-reactive T cells (Th17) to the gut. Thus, both pathobionts and pathogenic T cells derived from oral inflammation and translocated to the gut can collectively cause exacerbation of intestinal inflammation [55]. Another study reported that inoculation of saliva from children with CD to germ-free mice led to the accumulation of inflammatory IFN-γ^+^ CD4^+^ TH1 cells in the intestinal lamina propria and enrichment of *Fusobacterium*, *Veillonella*, and *Klebsiella* spp. in the fecal microbiome of the inoculated mice [12]. It was reported that multidrug-resistant *Klebsiella* strains isolated from the saliva of IBD patients can colonize the gut of mice when the intestinal microbiota is dysbiotic, eliciting a high-grade inflammatory response in the gut of genetically susceptible hosts [12]. These observations provide strong evidence supporting the connections between the oral–gut axis, oral microbiome, and immune-mediated mechanisms in IBD pathogenesis.

By collecting oral samples, it has been possible to assess the health of the gut by utilizing the oral–gut axis. Since each person produces between 1 and 2 L of saliva each day, saliva has been considered a desirable biospecimen for sampling and a valuable source of disease biomarkers [56,57]. Utilizing saliva as a biofluid has several benefits, including a quick, simple, non-invasive, and affordable collecting procedure that is suited outside of the laboratory and does not require medical professionals. Even though it is still in its early stages, research on the oral–gut axis shows promise [58].

## 6. Oral Dysbiosis Link to Oral Diseases and Contribution to IBD

It is speculated that the microbiology of the oral cavity may be unique in IBD patients [59]. W.D. Miller, a pioneering oral microbiologist, postulated in the 1890s that bacteria in the mouth and their products might have a dramatic impact on several illnesses, both local and systemic, owing to dental bacteremia, which was dubbed “oral focal infection hypothesis” [5]. Even though the mouth is constantly exposed to a bombardment of host and environmental toxins, the oral microbiome in healthy people remains reasonably consistent over time. Given this, alterations in the oral microbiota profile may provide correlative insight into illness initiation, progression, and recurrence.

Numerous studies have demonstrated the link between oral flora imbalance and inflammation, and how this link may contribute to the development of systemic diseases (Figure 2) such as Alzheimer’s disease, diabetes mellitus, cardiovascular disease, preterm birth, and obesity as well as gastrointestinal disorders like colorectal cancer and IBD and autoimmune disorders like rheumatoid arthritis through bacteremia [60]. One key aspect of the oral–gut connection is the transmission of specific microorganisms from the mouth to the gut. The reciprocal flow of bacterial pathogens provides more evidence of commonalities between the oral and gut microbiomes [61]. Oral microbiome disruptions caused by poor oral hygiene or induced by excessive sugar intake may result in oral diseases such as dental caries, and gum diseases including periodontitis. The oral microbiome has been well characterized in terms of its role in oral diseases, but its members have also been implicated as contributing factors in a variety of extra-oral diseases [51,62].

Some pathogenic oral bacteria, such as *Fusobacterium nucleatum* and *Porphyromonas gingivalis*, have been detected in gut biopsies of IBD patients, suggesting a potential role in disease progression. Dysregulated host–oral microbiota interaction is crucial in the pathogenesis of many oral diseases. IBD patients also appear to have an increased risk for oral diseases such as dental caries and periodontitis [63]. Moreover, studies have shown that individuals with periodontal disease are at a higher risk of developing IBD. Periodontitis is an inflammatory oral disease with a high global prevalence and association with multiple health issues [11,56,64]. It is a progressive chronic inflammatory condition that can lead to localized bone loss. In patients with periodontitis, several systemic markers of inflammation are increased; thus, it may represent an indirect mechanism explaining how the oral bacteria participate in the pathogenesis of systemic diseases, such as IBD [52]. Furthermore, gut microbiota dysbiosis can be induced by periodontitis via salivary microbiota. In a previous study, saliva-sourced microbes were enriched in the fecal samples of patients with severe periodontitis. When saliva from the latter group of patients was orally administered to mice, enrichment of oral bacteria from *Porphyromonadaceae* and *Fusobacterium* was evident. This was accompanied with pathological changes in the gut, as the colon tissues showed significantly higher expression of proinflammatory cytokines, chemokines and tight junction proteins with low zonula occludens-1 expression and crypt depth [65]. Thus, it is important to investigate oral health, IBD activity, and their relation to oral microbiota dysbiosis.

### 6.1. Oral Microbiome Alterations in CD

Although the host–microbe interaction has been linked to the pathophysiology of CD in genetically predisposed hosts, little is known about oral microorganisms in CD. Recent microbiome research suggests that the translocation of oral bacteria to the gut leads to microbial dysbiosis, which is a hallmark of CD [5]. Few studies have looked specifically at the influence of CD on the oral microbiota and its relation to oral health [50]. A common extraintestinal sign of CD is oral pathology, such as angular cheilitis, linear ulcerations, mucogingivitis, cobblestoning of mucosa, and persistent mucosal swelling [66]. Oral signs found in CD patients imply a link between oral microbiota and such manifestations; nevertheless, little is known about the oral microbiota of CD patients [31]. Regardless of the presence of oral symptoms, the oral mucosa is an immunologically active surface with higher cytokine generation. It has previously been reported that particular oral bacteria, such as a subset of *Porphyromonas gingivalis*, *Streptococcus mutans*, *Fusobacterium nucleatum*, *Campylobacter concisus*, and *Klebsiella pneumoniae*, may aggravate inflammation in CD [47]. Ectopic colonization by these oral bacteria may break down the intestinal epithelial barrier, generate excessive release of inflammatory cytokines, disturb the host immune system, promote immunological escape, and create gut microbiota dysbiosis, ultimately exacerbating chronic intestinal inflammation. Previous research focused only on changes in the makeup of the oral microbiota while ignoring the relationship between oral microbiota dysbiosis and the inflammatory state in the gut [47]. Although the makeup of the communities in the mouth and gut differs, the amount of species richness in both settings is comparable, and a single individual may house over 100 unique species at each site [47].

A significant reduction in both total microbial diversity and particular phylum levels was found in CD. Furthermore, the loss of certain phyla, such as *Fusobacteria* and *Firmicutes*, has been demonstrated in investigations of the gut microbiome in CD. The oral microbiota is changed in IBD patients, particularly in CD [59]. Remarkably, patients with CD and oral symptoms had significantly greater anti-*Saccharomyces cerevisiae* antibody (ASCA) titers than those without oral signs [59]. Salivary microbiota dysbiosis is associated with inflammatory responses in IBD patients, indicating that it is probably related to gut microbiota dysbiosis [47].

In a recent study, the differences in taxonomic and predicted functional pathways were clearly observed in saliva samples collected from patients with CD during active and remission phases of the disease compared to healthy controls. Both alpha and beta diversities were significantly lower during the active phase in contrast with the remission phase and healthy controls [27]. A total of 30 bacterial taxa were significantly enriched in the active phase. In contrast, remission phase and healthy controls exhibited significant enrichment of other 24 and 22 bacterial taxa, respectively. It has become evident that typical mouth-resident bacteria, such as *Fusobacteriaceae*, *Pasteurellaceae*, and *Veillonellaceae*, are enriched in the mucosal tissues of CD patients [28]. Another study reported that the salivary microbiota in CD patients was significantly different from that of healthy controls [31]. The relative abundance of *Bacteroidetes* was significantly higher, while that of *Proteobacteria* was significantly lower in the saliva of CD patients compared to the healthy controls. Several important pathways, such as ribosome biogenesis and energy metabolism, were depleted in the active phase. Eleven differentially abundant pathways were also identified; four were significantly enriched in healthy controls, whereas another seven were significantly enriched in the active phase of the disease. This study has highlighted several taxa and functional categories that could be implicated with the onset of CD and thus have the potential to serve as biomarkers of the active disease [27].

A substantial reduction in the total variety of tongue microbiota was seen in CD, according to a pediatric study that included 40 children with CD and 43 control patients without IBD. A phylum-level study of salivary microbiota showed that individuals with IBD had higher concentrations of *Bacteroidetes* and lower concentrations of *Proteobacteria*. These investigations have revealed oral microbial variations in individuals with IBD, suggesting the importance of the oral microbiome in IBD diagnosis and patient monitoring [67].

### 6.2. Oral Microbiome Alterations in UC

Numerous studies have examined the modifications in the microbiome of patients with UC. There have been reports of, among other variations, a decline in biodiversity and depletion of the phyla *Firmicutes* and *Bacteroidetes.* Changes in microbial composition have an impact on the amount of butyrate and other metabolites like H_2_S produced in the gut as well as the metabolites that are created there because of microbial activity [68].

Although oral lesions have sporadically been linked to UC, a recurrent pathology with a complicated etiology, little is known about the general makeup of the oral microbiome in UC patients or its significance in the pathogenesis of the illness. The oral microbiomes of healthy people and UC patients were compared to determine any potential changes in the oral microbial communities related to UC. For this, 16S rRNA gene sequencing was used to examine the salivary microbiota of 10 patients with UC diagnosed in the active period and 11 healthy controls [69]. A metataxonomic study of the oral core microbiome in UC patients showed a decline in alpha diversity and an imbalance in the relative proportions of some important members. In addition, four distinct species or phylotypes of *Staphylococcus* and other organisms were only found in UC patients and were undetectable in healthy individuals. A comprehensive picture of the existence of oral dysbiosis related to UC and the potential existence of oral biomarkers was shown before [69].

Using Illumina sequencing of the V3-V4 region of the 16S rRNA gene, the salivary microbiomes of 54 UC patients, 13 CD patients, and 25 healthy controls were compared. Comparing taxa abundances showed enrichment of *Streptococcus* and *Enterobacteriaceae* in UC and enrichment of *Veillonellaceae* in CD, with depletion of *Lachnospiraceae* and *Prevotella* in UC and depletion of *Neisseriaceae* and *Haemophilus* in CD [5].

In comparison to healthy people, the detection frequency of the specified species was noticeably greater in UC patients. Colitis was made worse by administering the specific *Streptococcus mutans* strains that were identified in patients. One potential risk factor for the worsening of UC is infection with some strains of very pathogenic *S. mutans* [70]. Only in gingivitis areas, UC patients had a significantly higher abundance of *S. aureus* and *P. anaerobius* than controls. *Peptostreptococcus anaerobius*, *Staphylococcus aureus*, *Streptococcus anginosus*, *Streptococcus intermedius*, *Streptococcus mitis*, *Streptococcus mutans*, and *Treponema denticola* were among the bacteria with different counts between the groups, according to a multiple comparison analysis. In either gingivitis or periodontitis areas, CD patients had significantly greater amounts of these microorganisms than UC patients. Only in gingivitis areas did UC patients have greater concentrations of *S. aureus* and *P. anaerobius* than controls. IBD patients had increased concentrations of opportunistic infection-related bacteria in inflammatory subgingival regions, which could be detrimental to the vital microbe–host connection [71].

Table 1 summarizes the studies exploring the oral microbiome in CD and UC patients, with microbiota alteration reported in each group. Indeed, fewer studies were conducted in the pediatric population compared to the adult population.

## 7. Factors Influencing Microbiome Composition and Their Impact on IBD Pathogenesis

The microbiota benefit from their mutualistic interaction with the human host while living in a nutrient-rich environment [76]. Indeed, many factors can affect the residing microbiota. Studies have shown that a diet missing essential nutrients needed for commensal survival, the use of drugs, physical exercise, smoking, and mental and emotional stress are some environmental factors that may lead to intestinal inflammatory disorders [58]. We summarized the most important factors contributing to microbiota dysbiosis in relation to IBD as follows.

### 7.1. Early Life Factors

During the first three years of life, the gut microbiome is gradually established [77]. Previous studies have shown that alterations in the gut microbiome in infants are associated with gastrointestinal disorders and other long-term health issues [78]. A few years after birth, the gut microbiota progressively takes shape to reach its mature composition, with temporal variations in the richness and order of dominating species being mostly influenced by food and host physiological conditions [31]. Early childhood “healthy” gut microbiota are necessary for microbiota–host interactions that have a long-lasting effect on adult health and disease status [6].

Some of the most crucial factors influencing the colonization of the early-life microbiome are antibiotic use, feeding habit, and mode of delivery [79]. In fact, early-life antibiotic exposure may potentially be a significant risk factor for developing IBD later in life. Moreover, it is also possible that prolonged use of broad-spectrum antibiotics, not just in early life, may be associated with the development of IBD [79]. Breastfeeding, especially prolonged breastfeeding, was preventative of the development of CD or UC. Bioactive compounds and immunological cells found in human breast milk serve important roles in forming the newborn immune system as well as passive immunity. Additionally, human milk oligosaccharides function as prebiotics, encouraging beneficial intestinal flora. Numerous research studies examined the relationship between the manner of delivery and IBD. These studies produced contradictory findings, with the majority of the studies finding no relationship. Although studies have indicated that Cesarean section causes distinct microbial community structure and function, these variations vanish within 6 weeks of age. C-sections may, thus, not be a sufficient prenatal risk factor for IBD [79]. Although research has been performed on genetically vulnerable animal models to assess the impact of particular environmental conditions on immune responses and microbiota, research on human subjects is very few. These linkages will be clarified in the future by human research intended to assess the effects of environmental variables on microbiota–immune interactions in individuals with established genetic risk factors for IBD [80].

### 7.2. Effect of Diet and Supplements

Indeed, gut dysbiosis can be induced or aggravated by the intake of some dietary components. Based on several case–control dietary studies, the link between a high-fat diet (HFD) and IBD is now well established, and an HFD is seen as a possible risk factor for developing IBD. Higher intestinal permeability and altered gut flora may contribute to the increased risk brought on by the HFD [76]. A Western diet is thought to play a significant role in both the obesity pandemic and the development of IBD; thus, it is not unexpected that Westernized nations have the greatest prevalence of IBD [58]. In contrast, high intakes of fibers from vegetables and fruits were linked to a lower risk of UC and CD [80]. While *Bacteroides* species are more prevalent in children who eat Western diets high in fat and poor in fiber, *Prevotella* and *Succinivibrio* are more prevalent in diets high in plants and fiber. Western diets rich in fat may have an influence on the development of inflammatory bowel illness by reducing biodiversity and SCFA production [50].

Additionally, a diet rich in heme (such as red meat and beef) is characteristic of the Western diet with a distinct impact on gut microbiota, increasing the risk of IBD [81,82,83]. Research on mice has shown that a diet rich in heme can cause disturbance of the gut homeostasis due to the overgrowth of pathogenic bacteria (*Proteobacteria*, especially *Enterobacteriaceae*), depletion of useful microbial groups (*Firmicutes*), decrease in essential SCFAs (butyrate) and a drop in the alpha diversity. This is accompanied by intense intestinal inflammation, which is very similar to the chemically induced colitis (dextran sodium sulfate; DSS) used in animal models of IBD [84].

Iron deficiency anemia is one of the complications of IBD due to intestinal bleeding, poor absorption caused by gut ulceration, and loss of appetite leading to less dietary intake of natural sources of iron. As iron supplementation may be needed in IBD patients with severe anemia, it must be administered with caution [85]. Oral iron administration may not be well tolerated in many patients and can have a negative impact on the gut and the microbiome, thus triggering inflammation and contributing to dysbiosis. This was proven experimentally in DSS-induced colitis, whereby administration of dietary iron exacerbates colitis. This was explained by the ability of iron to induce oxidative stress through the Fenton reaction, coupled with inflammation due to the activation of the nuclear factor kappa-B (NF-κB) pathway leading to upregulation of the expression of proinflammatory cytokines [86,87]. Another study proved the harmful effect of iron on the gut microbiome in DSS-induced colitis, in which a high dose of dietary iron caused a significant depletion of fecal *Firmicutes* and *Bacteroidetes*, concomitant with enrichment of *Actinobacteria* and *Proteobacteria.* Furthermore, a significant increase in fecal calprotectin level was noted, reflecting more intense inflammation correlated with increased disease activity in colitis [85]. Moreover, the harmful effect of oral administration of iron was shown in patients with IBD. A clinical trial was conducted to compare the effect of oral versus intravenous (IV) iron replacement therapy on the gut microbiome of IBD patients. However, both oral and IV therapies ameliorated iron deficiency, oral iron therapy induced gut dysbiosis due to the depletion of beneficial microbiota, namely *Faecalibacterium prausnitzii*, *Ruminococcus bromii*, *Collinsella aerofaciens*, and *Dorea* species [88,89].

On the other hand, there are some therapeutic modalities that rely on exclusive enteral nutrition (EEN) to alleviate IBD symptoms [90]. EEN involves the use of a nutritionally complete liquid diet provided exclusively for up to 8 weeks instead of usual solids. The capability to employ EEN as a substitute for corticosteroids to produce remission in CD is unique to children with IBD, especially after the first diagnosis of the disease. EEN is a successful, non-pharmacologic method of inducing remission that also enables doctors to address any dietary requirements of the kid whose health has been impaired by an inflammatory condition. EEN is thought to modify the microbiota and create an anti-inflammatory environment in the gut while also aiding mucosal repair; however, the precise method by which it functions is yet unknown. This dietary intervention is an alternative for the treatment of children and young adults since it does not have the negative effects or immunosuppression associated with many other treatments [7].

Noteworthy, dietary modulation of the gut microbiome in IBD has shown promising results. For instance, the Crohn’s disease exclusion diet (CDED), which is a low-fat, high-protein diet, has been effective for managing children with mild to moderate CD, as well as for patients with an unfavorable response to biological therapy. Changes in microbial diversity, depletion of *Proteobacteria*, and enrichment of *Firmicutes*, particularly *Clostridiales*, were noted following the intake of this diet [91]. Furthermore, studies in mice have shown that a ketogenic diet (KD) and low-carbohydrate diet (LCD) can cause modulation of specific composition and function of gut microbiota and metabolites. KD dramatically increased the abundance of *Akkermansia* and *Roseburia* associated with improved immune responses and protective barrier functions. Moreover, significantly fewer inflammatory responses with less expression of inflammatory cytokines were shown following KD. On the contrary, LCD caused opposite effects compared to KD [92]. Another study in UC patients confirmed that consuming a low-fat, high-fiber diet helped to resolve both dysbiosis and gut inflammation with prolonged remission. This improvement was correlated with the increase in the abundance of beneficial microbiota such as *F. prausnitzii* after regular consumption of this diet. Interestingly, remission was also accompanied by an increase in fecal acetate levels, which is one of the beneficial SCFAs with anti-inflammatory effects [93].

### 7.3. Effect of Exercise

Exercise can increase the microbiome diversity; increase the *Bacteroidetes/Firmicutes* ratio, which may help to reduce weight, obesity-related diseases, and gut disorders; and stimulate the growth of bacteria that can control mucosal immunity and improve barrier functions. Particularly, the *Firmicutes* phylum has more diversity, which supports the maintenance of a healthy intestinal environment [94]. Exercise altered the makeup of the gut microbiota in one study in which women engaged in physical activity to at least the level advised by the WHO, and 11 genera were substantially different between active and inactive women. The healthier bacteria *Faecalibacterium prausnitzii*, *Roseburia hominis*, and *Akkermansia muciniphila* were more prevalent in the active women. Metagenomic analysis indicated taxonomic modifications, including an increase in *Akkermansia* and a decrease in *Proteobacteria*, in another 6-week endurance exercise research without dietary changes [95]. However, increasing the training load (i.e., lengthening the exercise period or raising the level of physical activity) may lead to gut dysbiosis and have a detrimental impact on the digestive system and result in symptoms including nausea, vomiting, diarrhea, colic, flatulence, and stomach discomfort [96].

The role of exercise in preventing and restoring gut dysbiosis in patients with IBD is well established [97]. Physical exercise was thought to be a modulator of the intestinal microbiome, leading to positive clinical outcomes in IBD patients. Research has suggested that crosstalk between muscles and the gut microbiota is induced by exercise through the 5′ adenosine monophosphate-activated protein kinase and fasting-induced adipose factor pathways as well a reduction in fecal bile acids, an increase in SCFAs production, an increase in gut luminal IgA, a reduction in luminal transit time, and the activation of the stress hypothalamic–pituitary–adrenal axis [97].

### 7.4. IBD Drugs and Microbiota

Since there is currently no medication that may cure IBD, treatment focuses on symptom management, mucosal healing, inducing and maintaining remission of the active illness, preventing relapses, hospitalizations, and surgeries, and generally enhancing the patient’s quality of life [98]. Management of CD is decided by considering several factors, including the patient’s age, disease location, and behavior [99]. Most current therapies for IBD rely on suppressing the host immune system without direct effect on the microbes that cause or contribute to inflammation. Complete remission induced by these therapies occurs in less than half of patients; thus, discovering new therapeutic targets is needed [100]. Antibiotics, anti-inflammatory agents such as corticosteroids, and 5-aminosalicylic acid (5-ASA) are among the medications used to treat IBD. Corticosteroids are not always effective in maintaining remission and usually contribute to growth impairment in pediatric patients [101]. Immunosuppressive medications such as thiopurines are commonly used for the maintenance of remission in steroid-dependent and steroid-refractory IBD [102]. Immunomodulators are used for the maintenance of remission in moderate to severe IBD [4]. Biological therapies, including monoclonal antibodies such as anti-TNF (IFX, adalimumab, and certolizumab pegol), anti-integrin (VDZ), and anti-interleukin ustekinumab (blocking p40 subunit of IL12/23), are used to suppress the immune response. They are usually considered in IBD patients with high disease activity, features indicating poor prognosis, and inadequate response to standard therapy [99]. The widespread use of biological therapies and immunomodulators in pediatric patients has altered the natural course of the disease and impeded the progression of complications. The introduction of therapeutic monoclonal antibodies directed against TNF, a major proinflammatory pathogenic cytokine in IBD, has revolutionized the treatment of CD. The use of TNF-α antagonist (infliximab) was found effective in inducing and prolonging remission in children with IBD, helping in mucosal healing in this population, and reversing growth failure in children with severe refractory CD [103]. Interestingly, it has been reported that initiation of medical treatment with anti-TNF-α in pediatric patients with active IBD caused changes in the fecal microbial composition [104]. The abundance of six bacteria, including *Eubacterium rectale* and *Bifidobacterium* spp., predicted the response to anti-TNF-α medication. Another study demonstrated that the change in the treatment regimen influenced the gut microbiome of IBD patients, including those with CD [32]. Patients who had received a course of oral corticosteroids at disease flare had more microbiome fluctuations than patients on stable medications [32]. These findings indicate that the dynamics of the gut microbiome composition can be influenced by the medications used to treat the disease.

As for the oral microbiome, subgingival microbiota was investigated in pediatric CD patients. About 17 genera were reported as candidate biomarkers for the disease. Of them, *Capnocytophaga*, *Rothia*, and TM7 were more abundant in CD compared to healthy controls. *Alloprevotella*, *Fusobacterium*, *Porphyromonas*, and *Prevotella* were consistently decreased in the antibiotic-exposed CD patients compared to CD subjects not using antibiotics. CD-associated genera were not present in samples obtained after 8 weeks of treatment (either with corticosteroids, immunomodulators, enteral therapy, or 5-ASAs), suggesting alterations in CD microbiome community structure in response to successful therapy [74].

## 8. Microbiome Modulation to Treat IBD

Indeed, any treatment utilizing gut microbiota relies on the reversal of its dysbiosis. Addressing dysbiosis often involves restoring a healthy balance of the microbiome [19]. Microbiome-based personalized treatments are being explored to target specific imbalances or dysfunctions within microbial communities. This can be achieved through interventions such as dietary modifications, probiotic supplementation, prebiotics, postbiotics and lifestyle changes that support a diverse and balanced microbial community.

Probiotics are living non-pathogenic microbes administered in food or dietary supplements to offer health benefits to the host. Several microorganisms, such as *Lactobacillus*, *Bifidobacterium*, *Saccharomyces*, and *Streptococcus*, have been recognized with useful probiotic properties [105]. These microbes have multiple benefits to the gut via modulation of microbiota composition, modulation of the immune response, and enhancement of intestinal barrier functions [106]. In inflamed mucosa of active IBD or experimental UC, downregulation of the expression of proinflammatory cytokines like TNF-α, IL-6, and IFN-γ, nitric oxide synthase, and matrix metalloproteinase activity was shown after probiotic treatment [107]. Moreover, the secretion of vitamins and other metabolites by probiotic bacteria can indirectly modulate intestinal microflora, which can be induced to produce beneficial SCFAs, ultimately leading to the alleviation or improvement of IBD [108]. A recent study reported the use of programmable probiotics to modulate inflammation and gut microbiota for IBD treatment. In the latter study, effective oral delivery of genetically engineered bacteria (*Escherichia coli* Nissle 1917) that overexpress catalase and superoxide dismutase was used to clear reactive oxygen species in the inflamed tissue in mice. This method was successful in restoring intestinal homeostasis by improving the abundance of *Lachnospiraceae*_NK4A136 and *Odoribacter* in the intestinal flora. Treated mice showed rapid recovery of body weight and alleviated colonic mucosa damage with less colonic MPO activity. Moreover, probiotic effectiveness was proven in human model of IBD, more in UC than in CD [108]. Most of these studies reported improved symptoms, reduced recovery time, maintaining remission and preventing recurrence, and reduction in disease activity index and intestinal permeability. This was coupled with the cessation of inflammation marked by lower IL-1β expression and higher expression of IL-10 and IgA in the colonic mucosa [108]. Nevertheless, a few studies reported no therapeutic effect, as reported in one of the old studies showing the ineffectiveness of *Lactobacillus johnsonii LA1* to prevent postoperative recurrence in CD [109]. There has been a significant improvement in patient symptoms in several trials looking at probiotic supplements for the treatment of IBD. Numerous studies have shown the benefits of certain probiotic bacteria, including strains from the genera *Lactobacillus* and *Bifidobacterium.* Researchers discovered a reduction in inflammation because of metabolic changes resulting from altered gut microbial populations in a trial using fermented milk products containing *Bifidobacterium lactis* in mouse models of colitis. According to these data, a change to the gut environment with a more powerful commensal bacterial population may protect the gut lining and make it unfavorable for opportunistic strains [58]. According to the American Gastroenterological Association Institute, probiotics may be used to alleviate functional symptoms of IBD. By preventing the growth of harmful bacteria and encouraging the growth of suitable species, probiotics may have anti-inflammatory effects, enhance barrier function, and positively regulate the microbiome composition. In individuals with CD and UC, the probiotic cocktail VSL#3 (a combination of one strain of *Streptococcus*, plus three strains of *Bifidobacteria*, and four strains of *Lactobacilli*) decreased recurrence and maintained remission [33].

Postbiotics are composed mainly of SCFAs, enzymes, peptides, vitamins, peptidoglycans, and polysaccharides produced from microbial fermentation [110]. Among them, SCFAs are the most promising adjuvant therapy in the clinical management of IBD patients with active disease. Acetate, propionate, and butyrate were used either individually or in combination, mainly in patients with UC. The results were inconsistent as some studies reported beneficial effects and improvement in the clinical parameters, while others could not find any significant improvement [111]. Nevertheless, a recent study reported a better modulatory effect on the gut microbiome induced by postbiotics than probiotics (from *Bifidobacterium adolescentis* B8589) in a mouse colitis model [112].

Other microbial-based therapies for IBD include fecal microbiota transplantation (FMT), which is a technique of transferring healthy fecal microbiota to the gut of patients with dysbiosis-related gut disorders in order to restore a healthy gut microbiome. A large body of evidence proved the efficacy of FMT for the treatment of recurrent *Clostridium difficile* infection, but the effect was variable depending on the delivery method and the frequency of administrations [113]. In terms of clinical remission, frozen fecal material produced better results compared to fresh material. FMT can be delivered by oral capsules, enemas, nasogastric or nasojejunal tubes [107]. Current evidence on the effectiveness of FMT for IBD is inconclusive, according to a recent systematic review and meta-analysis [114]. However, there are studies supporting the use of FMT coupled with other treatment modalities. In a recent a randomized controlled trial, FMT provided with an anti-inflammatory diet followed by an anti-inflammatory diet alone was effective in inducing and maintaining remission over one year in cases of UC with mild to moderate disease [115]. Another recent randomized, double-blind, placebo-controlled trial reported that the administration of antibiotics followed by oral FMT was associated with the induction of remission in patients with active UC. Furthermore, continuing FMT was well tolerated, with better clinical, endoscopic, and histological scores [116]. There are reports proving the effect of FMT on gut microbiota restoration, mainly in UC. Several genera, such as *Bacteroides*, *Proteus*, and *Prevotella*, were significantly enriched, while pathogenic bacteria from the genera *Klebsiella* and *Streptococcus* decreased significantly after FMT [107].

Noteworthy, the vast majority of the previous studies had significant limitations related to the low sample size and difficulty in having proper study groups; for example, some patients may have concomitant treatment with immunosuppressants and steroids, which can affect outcomes [111]. This indeed pinpoints the need for conducting more comprehensive studies in the future.

## 9. Summary and Outlook

The microbiome encompasses a vast ecosystem of microorganisms that profoundly influence the health of their hosts [31]. Dysbiosis is the consequence of disruptions in the microbiome, which is described as changes in the organization of a microbial community. The resulting dysbiosis manifests as an altered balance of microbiota elements. This may impede key microbiome functions, including resistance to harmful bacteria. The study of the microbiome in different niches has greatly advanced our understanding of various diseases, including IBD. Dysbiosis in IBD is not limited to the gut but also includes the oral microbiome. Indeed, the oral–gut connection is a crucial component of IBD pathogenesis. Bidirectional communications between these two environments have implications for human health. Since the oral and gut microbiomes account for the majority of the overall human microbial load, they offer unique prospects for improving human health, diagnosis, prognosis, outcome prediction, and discovery of targeted therapies [61]. Further understanding of these microbiomes and their interactions with the human body has the potential to revolutionize healthcare. Renewed research efforts employing next-generation sequencing for high-resolution characterization of the composition, function, and ecology of microbiota have improved our overall knowledge of the role of microbiota in health, which is required for the study of disease-related dysbiosis.

Delving into the mechanisms of microbial interaction and their implications for disease pathogenesis indeed contributes to the growing body of knowledge in the field. Ultimately, a better understanding of the oral–gut microbiome relationship in IBD can pave the way for targeted interventions and personalized approaches to improve gut health and disease outcomes for affected individuals. Elucidating the intricate interplay between the oral and gut microbiome in IBD patients holds promise for novel therapeutic interventions. Strategies that target the oral microbiome, such as improved oral hygiene practices and periodontal disease treatment, may help in modulating the gut microbiome and alleviating IBD symptoms. Furthermore, manipulating the gut microbiome through dietary interventions, prebiotics, probiotics, and FMT could have a beneficial impact on oral and gut health.

## Figures and Tables

**Table 1:** Oral microbiome alterations in CD and UC patients.

Sample Type	IBD Type	Age Group	Sex	Sample Size	Geographical Region	Bacteria	Finding: Increased/Decreased	Reference
Saliva	CD	Adults	Sex was not considered—not mentioned	2	Japan	*Klebsiella*, *Fusobacterium*, and *Veillonella*	Increased	[12]
Saliva	CD	Adults	34 males and 31 females	65	Singapore	*Actinobacteria* and *Proteobacteria*	Increased	[72]
						*Firmicutes*, *Bacteriodetes*	Decreased	
Saliva	CD	Adults	392 males and 276 females	668	North America	*Fusobacterium nucleatum*, *Heamophilus parainfluenzae*, *Veillonella parvula*, *Eikenella corrodens*, and *Gemella moribillum*	Increased	[28]
						*Bacteroides vulgatus* and *Bacteroides caccae*	Decreased	
Saliva	CD and UC	Adults	38 males and 21 females	59	Japan	*Neisseria (phy. Proteobacteria.)*, *Gemella (phy. Firmicutes)*, *Proteobacteria*, *Neisseria*, and *Haemophilus*	Decreased	[31]
						*Bacteroidetes* and *Prevotella*	Increased	
Saliva	CD	Adults	57 males and 34 females	91	China	Phyla: *Firmicutes*, *Bacteroidetes*, and *Proteobacteria* Genera: *Streptococcus*, *Neisseria*, *Prevotella*, *Haemophilus*, and *Veillonella*	Increased	[27]
Saliva	CD and UC	Adults	14 males and 12 females	26	Germany	Phyla: *Fusobacteria*, *Proteobacteria*, and *Patescibacteria* Genera: *Neisseria*, *Streptococcus*, *Haemophilus*, *Porphyromonas*, and *Fusobacterium*	Decreased	[73]
						Phyla: *Firmicutes*, *Bacteroidetes*, and *Actinobacteria* Genera: *Veillonella* and *Prevotella*	Increased	
Saliva	UC	Adults	Sex was not considered—not mentioned	21	Spain	*Staphylococcus* and *Neisseria*	Increased	[69]
						*Peptostreptococcaceae*, *Atopobiaceae*, *Lachnospiraceae*, and *Ruminococcaceae*	Decreased	
Saliva	UC	Adults	Sex was not considered—not mentioned	92	China	*Streptococcus* and *Enterobacteriaceae*	Increased	[5]
						*Lachnospiraceae* and *Prevotella*	Decreased	
	CD					*Villanella*	Increased	
						*Neisseriaceae* and *Haemophilus*	Decreased	
Saliva	CD	Adults	18 males and 13 females	30	China	*Saccharibacteria (TM7)*, *Absconditabacteria (SR1)*, *Actinobacteria*, *Bulleidia*, *Parvimonas*, and *Prevotella*	Increased	[47]
						*Rothia*, *Corynebacterium*, and *Mycoplasma*	Decreased	
	UC					*Saccharibacteria (TM7)*, *Absconditabacteria (SR1)*, *Actinobacteria*, *Leptotrichia*, and *Atopobium*	Increased	
						*Rothia*, *Corynebacterium*, and *Mycoplasma*	Decreased	
Subgingival plaque samples	CD	Adults	22 males and 23 females	45	Brazil	Periodontitis sites: *Bacteroides ureolyticus*, *Campylobacter gracilis*, *P. melaninogenica*, *S. aureus*, *S. anginosus*, *Streptococcus intermedius*, *S. mitis*, and *S. mutans* Gingivitis sites: *Parvimonas* *micra*, *Prevotella melaninogenica*, *Peptostreptococcus anaerobius*, *Staphylococcus aureus*, *Streptococcus anginosus*, *Streptococcus mitis*, *S. mutans*, and *Treponema denticola*	Increased	[71]
	UC					Periodontitis sites: *Bacteroides* *ureolyticus*, *Campylobacter gracilis*, *P. melaninogenica*, *S. aureus*, *S. anginosus*, *Streptococcus intermedius*, and *S. mutans* Gingivitis sites: *P. anaerobius* and *S. aureus*	Increased	
						Gingivitis sites: *P. micra*, *S. anginosus*, and *S. mitis*,	Decreased	
Tongue and buccal mucosal brushings	CD	Pediatrics	62 males and 52 females	114	USA	*Fusobacteria* and *Firmicutes.*	Decreased	[59]
	UC					*Fusobacteria*	Decreased	
						*Spirochaetes*, *Synergistetes*, and *Bacteroidetes*	Increased	
Subgingival plaque samples	CD	Pediatrics	Sex was not considered—not mentioned	156	USA	*Alloprevotella*, *Campylobacter*, *Catonella*, *Fusobacterium*, *Porphyromonas*, *Prevotella*, *Selenomonas*, and *Veillonella*	Decreased	[74]
						*Capnocytophaga*, *Rothia*, and *TM7.*	Increased	
Tongue and buccal mucosal swabs	CD	Pediatrics	Male:female ratio: 2.6:1 in IBD 2:1 in healthy control	248	Ireland	*Prevotella*, *Fusobacterium*, *Leptotrichia*, *Rothia*, *Porphyromonas Veillonella*, *Oribacterium*, *Peptostreptococcaceae*, and *Lachnoanaerobaculum*	Decreased	[75]
						*Lachnospiraceae*, *Oribacterium*, *Catonella*, *Stomatobaculum*, and *Ruminococcaceae*	Decreased in association with severe IBD	
						‘IBD-associated’ taxa *Eikenella* and *Pseudopropionibacterium* spp.	Decreased after therapy	
						*Ottowia*, *Pseudopropionobacterium*, *Lautropia*, *Staphylococcus*, *Pseudomonas* and *Corynebacterium species*, *Eikenella*, and *Streptococcus species.*	Increased	
						*Lactobacillus*, *Streptococcus*, *Staphylococcus*, and *Klebsiella* spp.	Increased in those with severe IBD	
						‘Health-associated’ taxa: *Veillonella* spp. and *Oribacterium* spp.	Increased after therapy	

## Data Availability

Not applicable.
